# Exploring early perceptual processing of faces displaying Down syndrome features from a masked priming paradigm

**DOI:** 10.3389/fpsyg.2026.1873993

**Published:** 2026-10-16

**Authors:** Nazaret Hernández-Espeso, Carmen Moret-Tatay, Loreto Bustos-Hernando, Roberta Diamanti

**Affiliations:** 1 Catholic University of Valencia San Vicente Mártir, Valencia, Spain; 2 University of Valencia, Valencia, Spain

**Keywords:** contextual priming, Down syndrome, early perceptual processing, face perception, intellectual disability, masked priming

## Abstract

**Background:**

Understanding how facial characteristics associated with intellectual disability are processed during early stages of perception may provide new insights into disability-related social cognition. Masked priming paradigms offer a useful approach for examining how contextual information influences face recognition before conscious deliberation.

**Methods:**

Digitally generated facial stimuli displaying Down syndrome characteristics were developed from standardized facial photographs and validated by 66 experts and 136 participants from the general population. The validated stimuli were subsequently used in a masked priming face recognition task completed by 22 psychology students, in which reaction times (RTs) and recognition accuracy were recorded across three priming conditions.

**Results:**

Priming condition significantly influenced reaction times, with faster responses observed in the identity condition, in which prime and target shared both identity and facial phenotype, than under feature-based or different priming conditions. Facial phenotype alone did not significantly affect reaction times or recognition accuracy. However, recognition accuracy varied according to the interaction between priming condition, facial phenotype, and stimulus type, indicating that behavioural performance depended on the experimental context rather than on facial phenotype alone.

**Conclusion:**

This exploratory study provides preliminary evidence that the recognition of faces displaying Down syndrome characteristics involves early perceptual processes. The findings contribute to the emerging field of disability-related social cognition and support the use of experimental cognitive paradigms to better understand disability perception.

## Introduction

Intellectual disability (ID) is characterized by significant limitations in intellectual functioning and adaptive behavior that originate during the developmental period and interact with environmental and contextual factors (Schalock and Luckasson, 2021). Although rights-based approaches and person-centered models have substantially improved the conceptualization of disability, people with ID continue to experience social exclusion and attitudinal barriers that restrict participation across educational, healthcare, and community settings (Friedman, 2020a, 2020b; Verdugo, 2025). These barriers may arise not only from consciously endorsed beliefs but also from automatic cognitive processes that influence how individuals perceive and respond to members of stigmatized social groups.

One cognitive mechanism that may contribute to these barriers is implicit bias, defined as automatically activated associations or evaluations that influence perception, judgment, and behavior outside conscious awareness (Greenwald and Banaji, 1995; Amodio and Mendoza, 2010). Unlike explicit attitudes, which are consciously accessible and typically assessed using self-report measures, implicit biases are inferred from indirect behavioral paradigms designed to capture rapid, automatic responses (Antonopoulos et al., 2023). Importantly, implicit bias should not be equated with related constructs such as prejudice or discrimination. Whereas prejudice refers to negative evaluations toward a social group and discrimination concerns differential behavior, implicit bias reflects automatic associative processes that may occur independently of consciously endorsed beliefs (Amodio and Mendoza, 2010). Understanding these automatic processes is important because they may influence early stages of social perception even when explicit attitudes appear egalitarian.

Several paradigms have been developed to investigate automatic cognitive processes. The Implicit Association Test (IAT) is the most frequently used paradigm for investigating disability-related implicit bias and assesses the strength of automatic evaluative associations between social categories and attributes (Greenwald et al., 1998). Other paradigms, including affective priming, evaluative priming, and masked priming, examine different aspects of automatic information processing (Huang et al., 2025). A recent scoping review concluded that, although the IAT dominates disability research, alternative experimental paradigms remain comparatively underused despite offering valuable insights into early cognitive processing (Huang et al., 2025). Consequently, these paradigms should be considered complementary rather than interchangeable, as they capture different components of automatic cognition (Antonopoulos et al., 2023).

Among these approaches, masked priming paradigms provide an opportunity to examine early stages of perceptual processing before conscious awareness. Face perception is particularly suitable for investigating such processes because facial information is extracted rapidly and automatically, allowing observers to categorize socially relevant characteristics within a few hundred milliseconds (Bruce and Young, 1986; Haxby et al., 2000). Rather than directly measuring attitudes, masked priming paradigms examine how previously presented information influences subsequent perceptual processing under conditions of limited conscious awareness. In this context, reaction times and recognition accuracy provide indirect indicators of the efficiency of automatic perceptual processing. However, differences in these measures should be interpreted cautiously, as they may reflect multiple mechanisms, including perceptual familiarity, representational accessibility, or automatic associative processing, rather than direct evidence of prejudicial attitudes. Accordingly, the present study adopts masked priming as an indirect experimental paradigm for investigating context-dependent automatic perceptual processing that may be influenced by implicit associative mechanisms, rather than as a direct measure of implicit prejudice or ableist attitudes.

Despite increasing interest in disability-related implicit bias, research examining the automatic perceptual processing of faces displaying intellectual disability remains scarce. Most studies have focused on explicit attitudes or implicit evaluative measures such as the IAT, demonstrating the persistence of ableist attitudes among healthcare professionals, disability service providers, and members of the general population (Friedman, 2019, 2023; VanPuymbrouck et al., 2020). Furthermore, both the quantity and quality of interpersonal contact appear to influence these attitudes, with more frequent and meaningful contact being associated with lower levels of implicit bias (McConkey et al., 2021; Servidio et al., 2024). However, relatively little is known about whether these processes are reflected in the automatic perceptual processing of facial information, particularly among psychology students, who represent future professionals likely to interact with individuals with intellectual disabilities.

Face processing involves the rapid extraction of multiple levels of information. Moret-Tatay et al. (2021) distinguished relatively stable facial traits, including identity and socially relevant characteristics, from more transient facial states such as emotional expression, and emphasized that viewing a face rapidly engages categorization and identity-recognition processes. This distinction is particularly relevant to the present paradigm, in which the relationship between identity and phenotype-related facial characteristics was experimentally manipulated across priming conditions.

In this way, the masked priming paradigm was considered particularly appropriate for addressing the present research question, as it allows the examination of whether contextual information influences the early perceptual recognition of faces displaying Down syndrome characteristics before conscious evaluation. Recognition decisions may reflect either familiarity or retrieval of identity-specific information; in the present masked-priming paradigm, the prime was expected primarily to modulate perceptual familiarity/accessibility rather than deliberate recollection.

In a recognition task, a masked prime may influence performance by temporarily increasing the accessibility or familiarity of perceptual representations that overlap with the subsequently presented face. When prime and target correspond to the same identity and phenotype, this pre-activation should facilitate recognition because both identity-specific and phenotype-related perceptual information are congruent.

When a prime preserves the identity of the person while changing phenotype-related facial characteristics (as in the feature-based priming condition used in this study) a partial mismatch is created in which identity-relevant information may still be pre-activated, but changes in facial configuration may attenuate the facilitation associated with identity continuity. When the prime depicts a completely different individual, identity-specific correspondence is also absent, and facilitation should be further reduced.

This mechanism may be particularly relevant when recognizing neurotypical faces compared to recognizing faces displaying Down syndrome characteristics.

Observers are likely to have greater everyday perceptual experience with neurotypical faces, which may contribute to more differentiated and readily accessible identity representations. Consequently, an identity-congruent prime could produce a stronger familiarity or representational-accessibility benefit for neurotypical faces. Conversely, more limited perceptual experience with faces displaying Down syndrome characteristics could reduce the extent to which identity-specific pre-activation facilitates subsequent recognition. This interpretation concerns perceptual familiarity and representational accessibility rather than prejudice or evaluative bias and should be considered tentative because prior contact with people with Down syndrome was not directly assessed.

Accordingly, we expected recognition to be most efficient in an identity-prime condition, in which prime and target shared both identity and facial phenotype. Performance was expected to be reduced in a condition where identity was preserved but phenotype-related facial characteristics changed, because this perceptual mismatch could attenuate identity-based facilitation. The lowest facilitation was expected in a condition where the prime and target depicted different individuals. We further explored whether these priming effects differed between neurotypical faces and faces displaying Down syndrome characteristics. Given that participants’ previous perceptual experience with people with Down syndrome was not directly assessed, predictions regarding phenotype-related differences were considered exploratory.

## Materials and Methods

### Participants

The initial sample consisted of 30 undergraduate psychology students, aged between 18 and 33 years (*M* = 22.06, *SD* = 3.24). Eight participants were excluded due to technical errors or high failure rates, leaving a final sample of *n* = 22 for data analysis. Of these, 20 were women (91.8%) and 2 were men (8.2%). The study was approved by the Ethics Committee, and all participants provided written informed consent prior to participation.

### Stimuli

Neutral-expression images were selected from the Karolinska Directed Emotional Faces database (KDEF; Lundqvist et al., 2015). The use and modification of these stimuli complied with institutional ethical guidelines and did not require individual consent, as the images belong to an open-access repository validated for research purposes. The images were digitally manipulated using Adobe Express with artificial intelligence tools to simulate facial features associated with Down syndrome and Williams syndrome. The image editing process aimed to preserve each individual’s facial identity while introducing facial characteristics commonly associated with Down syndrome. Specifically, modifications focused on facial morphology, including eye shape and palpebral fissures, nasal bridge configuration, facial roundness, and overall facial proportions. The editing process was iterative and supervised by members of the research team with experience in intellectual disability research to ensure that the resulting images represented recognizable Down syndrome facial characteristics while avoiding exaggerated or stereotypical depictions. A total of 35 stimuli were created: 17 depicting Down syndrome features and 16 depicting Williams syndrome features. The manipulated images were used exclusively for research purposes and do not represent real individuals with disabilities. The editing procedure was carried out with full respect for the dignity and appropriate representation of persons with intellectual disabilities.

All images were standardized in size, lighting, and background to ensure homogeneous presentation conditions. The validity of these 35 stimuli was established in two phases: an initial sample of 66 professionals with expertise in intellectual disability evaluated the images, followed by a replication phase with 136 participants from the general population. Both groups rated each image on a scale from 0 (no disability) to 10 (evident disability). Based on these evaluations, the images representing Williams syndrome were excluded, and the 16 stimuli depicting Down syndrome (8 male and 8 female faces) were selected, as they obtained more consistent and differentiated mean scores in the perception of disability, thereby ensuring their discriminant validity.

### Procedure

The experiment was conducted in a controlled environment, within quiet rooms with homogeneous lighting. The images were presented on a monitor with a resolution of 1,024 × 768 pixels in full-screen mode. Participants were seated approximately 60 cm from the screen, a distance consistent with standards used in recent facial recognition studies (Bobak et al., 2019; Wang et al., 2023). This setup ensured an adequate visual angle for proper perception of facial features and maintained a perceptual stimulus size comparable to that used in similar experiments. The task lasted approximately 25 min. A mid-session break was included in the protocol, and participants decided when to resume the task, which helped minimize fatigue and ensure optimal performance throughout the session. The task consisted of a masked priming facial recognition paradigm, implemented using DMDX software (Forster and Forster, 2003). In the encoding phase, participants were instructed to memorize two target faces (one male and one female), corresponding either to the Down syndrome or the neurotypical condition. The faces were presented individually during a dedicated learning phase before the recognition task began. Participants were instructed to memorize the identity of both faces because they would subsequently have to decide whether each face presented during the experimental task corresponded to one of these previously learned target faces. Following the learning phase, the recognition task started immediately.

In the recognition phase, target and distractor faces were presented in random order under three priming conditions: identity, feature-based, and different. Participants were required to decide whether each presented face corresponded to one of the two previously memorized target faces (“target”) or to a new face (“distractor”), responding as quickly and accurately as possible.

Each trial followed the same sequence: (1) a fixation cross (+), (2) a briefly presented prime immediately followed by a mask, (3) presentation of the face for 2,500 ms, and (4) a response window. Participants responded by pressing the “M” key (with the right hand) if they recognized the face as a target, or the “Z” key (with the left hand) if they judged it to be a distractor. Response keys were not counterbalanced; all participants used the same configuration.

Because the study employed a fully within-subjects design, every participant completed all experimental conditions using the same response-key assignment. Consequently, any motor asymmetry associated with the fixed key configuration would have affected all experimental conditions equally and is therefore unlikely to account for the observed differences between experimental conditions. Each stimulus was repeated 14 times, distributed across counterbalanced lists to control for order and learning effects (see Figure 1).

**Figure 1:**
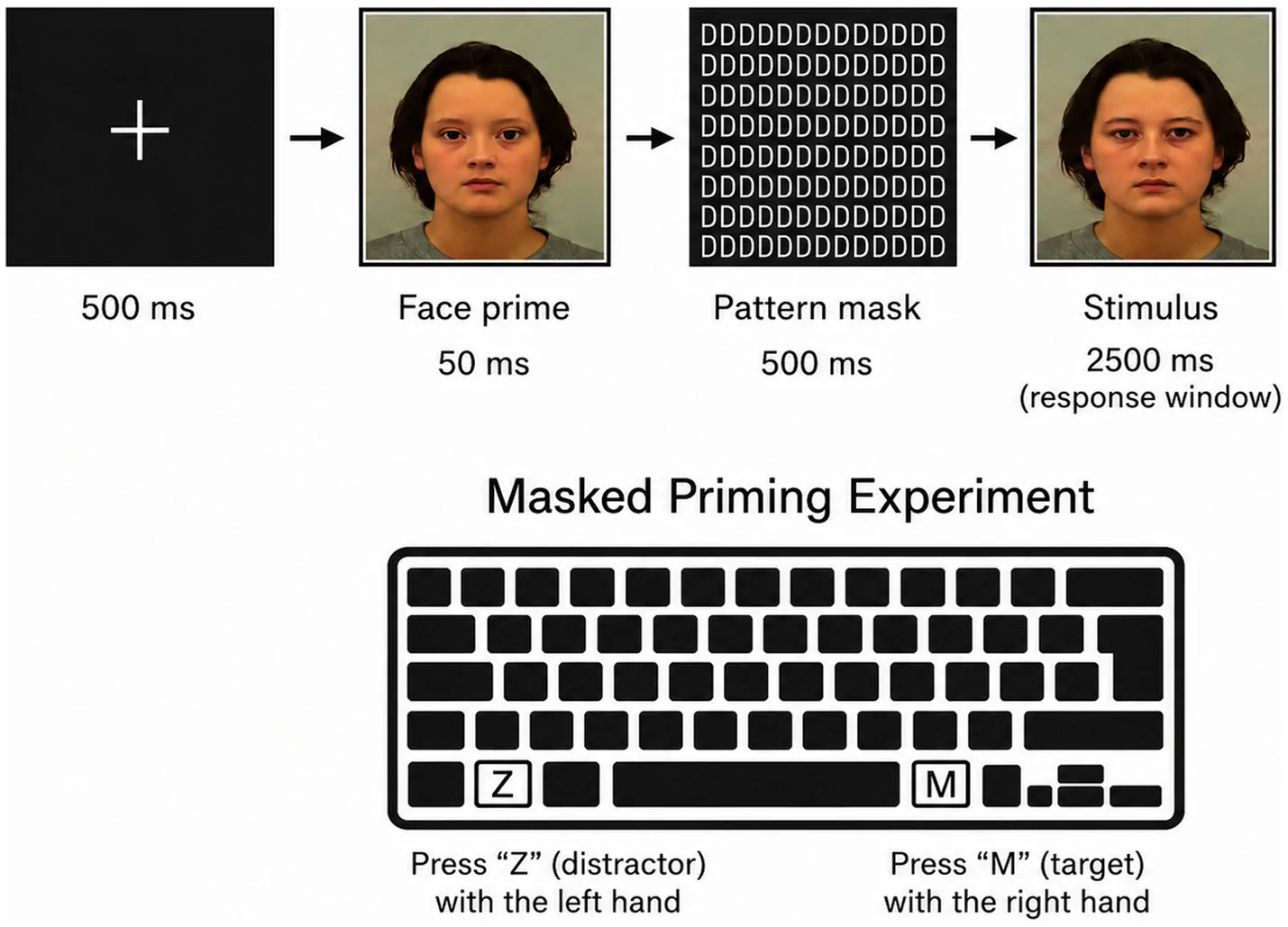
Diagram showing a masked priming sequence: fixation cross, face prime, mask, and face stimulus. Responses are made using Z for distractors (left hand) and M for targets (right hand). Face image from the Karolinska Directed Emotional Faces (KDEF; Lundqvist, Flykt, and Öhman, 1998), reproduced and adapted under the KDEF terms for scientific publication.

### Design and data analysis

A within-subjects design was employed with three main factors: Prime condition (identity, feature-based, different), Phenotype (Down syndrome vs. neurotypical), and Stimulus type (target vs. distractor). Thus, three priming conditions were used to manipulate the relationship between the prime and the subsequently presented face. In the identity condition, the prime and the stimulus depicted the same individual with the same facial phenotype. In the feature-based condition, the prime depicted the same individual but with a different facial phenotype from the subsequently presented stimulus. In the different condition, the prime depicted a different individual with a different facial phenotype from the stimulus, providing the lowest level of both identity-specific and phenotype-related correspondence between prime and stimulus.

The dependent variables were reaction times (RTs, in ms) and accuracy (percentage of correct responses). Only RTs from correct trials were included in the analyses. Trials with RTs < 250 ms or > 1,800 ms were excluded. Data were analyzed using repeated-measures ANOVA in SPSS version 23. The assumptions of normality and sphericity were verified, and the Greenhouse–Geisser correction was applied when necessary. Additionally, 95% confidence intervals (CIs) for the means were computed and reported along with the corresponding *F*, *p*, and partial eta squared (η^2^_p_) values as measures of effect size. Given the exploratory nature of the study and the relatively small sample, confidence intervals were considered particularly informative for interpreting the precision of the estimated effects.

## Results

### Descriptive Statistics

Table 1 presents the mean reaction times (RTs, in milliseconds), standard deviations (SDs), and accuracy rates (proportion of correct responses) for each combination of prime condition, facial phenotype, and stimulus type.

**Table 1:** Reaction times, standard deviations, and accuracy by condition.

Condition	Stimulus	Prime	RT (ms)	SD (ms)	Accuracy
Target	Normotypical	Identity	739.58	151.64	0.87
Target	Normotypical	Features	782.55	151.76	0.79
Target	Normotypical	Different	799.16	86.85	0.75
Target	Down	Identity	758.85	160.5	0.74
Target	Down	Features	800.82	118.54	0.81
Target	Down	Different	856.47	117.81	0.79
Distractor	Normotypical	Identity	748.22	116.79	0.8
Distractor	Normotypical	Features	802.28	102.67	0.8
Distractor	Normotypical	Different	844.36	106.44	0.81
Distractor	Down	Identity	736.43	111.93	0.85
Distractor	Down	Features	795.93	122.63	0.78
Distractor	Down	Different	814.09	97.76	0.79

### Reaction times

The repeated-measures ANOVA on reaction times revealed a significant main effect of prime condition, *F*(2, 42) = 12.04, *p* < 0.001, η^2^_p_ = 0.36. Bonferroni-corrected *post hoc* comparisons indicated that reaction times in the identity condition were significantly faster than in both the feature-based (*p* = 0.002) and different (*p* < 0.001) conditions, whereas the difference between the feature-based and different conditions was not significant (*p* = 0.118). Descriptively, participants responded faster in the identity condition (*M* = 739 ms) than in the feature-based (*M* = 791 ms) and different (*M* = 826 ms) conditions. Neither stimulus type (*F*(1, 21) = 0.01, *p* = 0.944, η^2^_p_ < 0.01) nor phenotype (*F*(1, 21) = 1.85, *p* = 0.188, η^2^_p_ = 0.08) showed significant main effects. No significant second- or third-order interactions were found (all *p*s > 0.05).

### Accuracy

The repeated-measures ANOVA on accuracy did not reveal significant main effects of prime condition, phenotype, or stimulus type (all *p*s > 0.05). However, two relevant interactions were observed. First, a prime × phenotype interaction, *F*(2, 42) = 6.36, *p* = 0.004, η^2^_p_ = 0.23, indicated that recognition accuracy varied by facial phenotype depending on the priming condition. For example, in the identity condition, neurotypical faces were recognized more accurately (*M* = 0.87) than faces with Down syndrome features (*M* = 0.74). Second, a significant three-way interaction among prime, phenotype, and stimulus type, *F*(2, 42) = 7.30, *p* = 0.002, η^2^_p_ = 0.26, was found, reflecting a more complex pattern of facial discrimination across experimental conditions. In particular, accuracy was higher for Down syndrome distractors (*M* = 0.85) than for Down syndrome targets (*M* = 0.74), whereas among neurotypical faces, the differences between targets and distractors were smaller.

Because the present study was exploratory and based on a relatively small sample, these significant interactions were interpreted at the omnibus level. Future studies with larger samples should further examine these interaction patterns using planned simple-effects analyses to better characterize the underlying relationships.

## Discussion

The present study examined whether facial phenotypes associated with Down syndrome influence face recognition and whether contextual priming modulates behavioural responses during early perceptual processing. The findings indicate that priming condition significantly affected reaction times, with participants responding faster when the prime and target shared both identity and facial phenotype than under feature-based or different priming conditions. In contrast, facial phenotype alone did not significantly influence either reaction times or recognition accuracy. However, recognition accuracy varied according to the combination of priming condition, facial phenotype, and stimulus type, suggesting that the effect of facial phenotype depended on the experimental context rather than representing a general difference in performance. In this way, the absence of a significant main effect of facial phenotype suggests that any influence of Down syndrome facial characteristics on behavioural performance was not sufficiently large or consistent to be detected as a general effect in the present sample.

The reaction-time pattern is particularly informative when the structure of the priming conditions is considered. Responses were fastest when prime and target preserved both identity and facial phenotype. In the feature-based condition, identity was preserved but phenotype-related facial characteristics changed, and responses were significantly slower than in the identity condition. Importantly, reaction times in the feature-based condition did not differ significantly from those in the different condition. Thus, preserving identity while altering phenotype-related facial characteristics did not produce a detectable reaction-time advantage over a condition in which both identity and phenotype differed. This pattern is consistent with the possibility that recognition depends not only on identity-specific information but also on the perceptual correspondence between the prime and the subsequently presented facial configuration.

In this regard, recognition accuracy varied according to the interaction between priming condition, facial phenotype, and stimulus type, suggesting that behavioural performance depended on the contextual relationship between the prime and the stimulus rather than on facial phenotype alone. This pattern highlights the dynamic nature of facial processing, in which contextual information and previously activated representations may influence recognition performance. Such findings are consistent with theoretical models of face perception proposing that facial recognition results from the interaction between perceptual analysis and stored identity representations rather than from isolated processing of facial features (Bruce and Young, 1986; Haxby et al., 2000).

Furthermore, these findings are broadly consistent with contemporary models of person perception, which propose that social categorization emerges through continuous interactions between incoming perceptual information and previously acquired knowledge (Freeman and Ambady, 2011). Rather than relying exclusively on structural facial cues, individuals rapidly integrate contextual information, prior experience, and associative representations when interpreting socially relevant stimuli. Within this framework, masked priming paradigms provide a valuable approach for examining these early automatic processes because they influence perception before conscious deliberation becomes fully engaged (Amodio and Mendoza, 2010).

Importantly, the absence of a significant main effect of facial phenotype should not be interpreted as evidence that disability-related information has no influence on perceptual processing. Instead, this null finding suggests that any potential influence of Down syndrome facial characteristics was neither sufficiently robust nor sufficiently consistent to emerge across all experimental conditions. Given the relatively small sample and exploratory nature of the present study, larger studies are needed to determine whether subtle effects of facial phenotype can be detected more reliably.

The accuracy results provide a more specific picture of this contextual modulation. In the identity-prime condition, neurotypical faces were recognized more accurately than faces displaying Down syndrome characteristics (0.87 vs. 0.74). Thus, identity priming did not produce an equivalent recognition benefit across facial phenotypes. Instead, the descriptive pattern was consistent with a stronger identity-priming benefit for neurotypical faces.

One possible explanation concerns perceptual familiarity and the accessibility of stored facial representations. Participants are likely to have substantially greater everyday experience discriminating among neurotypical faces than among faces displaying Down syndrome characteristics. Greater perceptual experience may produce more differentiated identity representations, allowing an identity-congruent prime to pre-activate the subsequent target more effectively and thereby increase familiarity at recognition. Conversely, less extensive experience with Down syndrome faces may result in less differentiated identity representations and therefore a weaker benefit from identity priming. This interpretation remains tentative because prior contact with people with Down syndrome was not measured in the present study.

The significant three-way interaction further indicates that this effect depended on whether the subsequently presented face was a previously learned target or a distractor. In particular, Down syndrome distractors were identified more accurately than Down syndrome targets (0.85 vs. 0.74), whereas target-distractor differences were smaller for neurotypical faces. This pattern suggests that the difficulty was not a generalized inability to process Down syndrome facial characteristics. Rather, it may have involved the formation or retrieval of sufficiently distinctive identity representations for previously learned Down syndrome targets. Future studies using larger samples and planned simple-effects analyses are needed to determine whether this pattern reflects reduced identity-specific familiarity, categorical processing, or another perceptual mechanism.

Although these findings are compatible with automatic associative processes, they should not be interpreted as either evidence for or evidence against implicit prejudice. The present results can be situated within the broader literature on disability-related social cognition. Recent research has shown that disability bias often operates through subtle and automatic mechanisms that influence expectations, decision-making, and interpersonal interactions even among individuals who explicitly endorse inclusive values (Friedman, 2023). Similarly, studies examining attitudes toward people with intellectual disabilities consistently report that greater opportunities for meaningful intergroup contact are associated with lower levels of prejudice and more positive attitudes (McConkey et al., 2021; Servidio et al., 2024). Although previous contact with people with intellectual disabilities was not measured in the present study, these findings suggest a plausible framework through which social experience may influence the accessibility of disability-related representations during rapid perceptual processing.

From this perspective, the current findings might be interpreted as evidence that contextual information contributes to behavioural responses during the recognition of faces displaying Down syndrome characteristics. Rather than demonstrating a generalized perceptual bias, the results highlight the importance of considering the interaction between perceptual context and socially acquired knowledge when investigating face processing in the context of intellectual disability. This distinction is particularly relevant because it avoids equating context-dependent perceptual modulation with implicit prejudice while acknowledging that both processes may share common associative mechanisms. Future studies incorporating larger and more diverse samples, ecologically valid facial stimuli, and complementary measures of implicit cognition will be essential for clarifying how perceptual, cognitive, and social processes interact in shaping responses to disability-related facial information.

Several limitations should be considered when interpreting the present findings. First, the study included a relatively small sample (*n* = 22), composed exclusively of university students. Furthermore, the sample consisted predominantly of women, preventing examination of possible gender-related differences in face recognition and limiting the generalizability of the findings to more gender-balanced populations.

Although this sample was appropriate for an exploratory experimental study, its size limits statistical power, particularly for detecting small effects, and restricts the generalizability of the findings to other populations. Replication with larger and more heterogeneous samples, including participants from different educational backgrounds and professional groups, is therefore warranted. Second, the interpretation of the null main effect of facial phenotype requires caution. The absence of a statistically significant effect should not be interpreted as evidence that facial phenotype has no influence on perceptual processing. Rather, it indicates that no consistent overall effect was detected under the present experimental conditions. Future studies employing larger samples and complementary statistical approaches, such as Bayesian analyses or equivalence testing, may help determine whether the observed null effect reflects a genuine absence of differences or limited statistical sensitivity.

Furthermore, some participant characteristics that could potentially influence performance in reaction-time paradigms were not systematically assessed. These included handedness, previous meaningful contact with individuals with intellectual disabilities, and neurological or psychiatric history. Although the within-subjects design and identical response-key assignment make it unlikely that handedness systematically influenced comparisons across conditions, future studies should include these variables to improve methodological control and to examine their possible moderating role in disability-related perceptual processing.

Lastly, the use of digitally generated facial stimuli should be considered when interpreting these findings. Although the images were carefully examined prior to the experiment, they cannot fully capture the natural morphological variability and subtle facial characteristics of real individuals with Down syndrome. Consequently, the present findings should be interpreted within the context of an experimental paradigm using controlled stimuli, and future studies should examine whether similar patterns emerge when using photographs of real individuals.

Beyond its exploratory findings, this study contributes to an emerging line of research at the intersection of cognitive psychology, disability studies, and inclusive education. While previous research has predominantly focused on explicit attitudes, stereotypes, and implicit evaluative associations, considerably less attention has been devoted to the perceptual processes through which disability-related information is initially encoded. By applying a masked priming paradigm, the present study provides a novel perspective on how contextual information may influence the recognition of faces displaying Down syndrome characteristics. Although these findings should not be interpreted as evidence of implicit prejudice, they suggest that early perceptual processes deserve greater attention within disability research. Importantly, this perspective also has educational implications. If perceptions of disability are shaped not only by explicit beliefs but also by contextual and experiential factors, higher education should move beyond the transmission of theoretical knowledge to provide meaningful opportunities for sustained contact and collaboration with people with intellectual disabilities. Recent participatory initiatives demonstrate that involving people with intellectual disabilities as educators, researchers, and collaborators enriches students’ learning while promoting more accurate and person-centred understandings of disability (Himmelberger et al., 2023; Sándor et al., 2024; Sándor et al., 2025; Otieno et al., 2025). Integrating cognitive research with inclusive educational practices may therefore contribute not only to advancing theoretical knowledge but also to preparing future professionals capable of recognising and responding to intellectual disability through evidence-based, inclusive, and person-centred approaches.

## Conclusion

The present exploratory study investigated whether contextual priming influences the recognition of faces displaying Down syndrome characteristics during early perceptual processing. The findings indicate that behavioural performance was shaped by the interaction between contextual information and facial phenotype rather than by facial phenotype alone, suggesting that face recognition is influenced by dynamic contextual processes. Although no overall effect of facial phenotype was observed, the significant interactions in recognition accuracy highlight the importance of considering the contextual conditions under which disability-related facial information is processed. These findings extend the literature on disability-related social cognition by introducing an experimental cognitive approach to the study of intellectual disability. Rather than focusing exclusively on explicit attitudes or stereotypes, the present study suggests that early perceptual mechanisms represent an additional level of analysis for understanding how disability-related information is processed. While these findings should not be interpreted as evidence of implicit prejudice, they support the value of integrating cognitive psychology with disability research to better understand the mechanisms underlying social perception. From an applied perspective, the findings also reinforce the importance of educational environments that promote meaningful and sustained interactions with people with intellectual disabilities. Such experiences may not only improve explicit attitudes but also contribute to shaping the contextual and experiential knowledge that underlies social perception. Future research using larger and more diverse samples, ecologically valid stimuli, and multimethod approaches will be essential to clarify these processes and to inform evidence-based educational and professional practices that foster inclusion and person-centred care.

## Data Availability

The raw data supporting the conclusions of this article will be made available by the authors, without undue reservation.
